# Atom size electron vortex beams with selectable orbital angular momentum

**DOI:** 10.1038/s41598-017-01077-9

**Published:** 2017-04-19

**Authors:** Darius Pohl, Sebastian Schneider, Paul Zeiger, Ján Rusz, Peter Tiemeijer, Sorin Lazar, Kornelius Nielsch, Bernd Rellinghaus

**Affiliations:** 1 IFW Dresden, Institute for Metallic Materials, Helmholtzstrasse 20, D-01069 Dresden, Germany; 2 TU Dresden, Institute for Solid State Physics, D-01062 Dresden, Germany; 3 Uppsala University, Department of Physics and Astronomy, SE-752 37 Uppsala, Sweden; 4 FEI Company, PO Box 80066, 5600 KA, Eindhoven The Netherlands; 5 TU Dresden, Institut für Werkstoffwissenschaft, D-01062 Dresden, Germany

## Abstract

The decreasing size of modern functional magnetic materials and devices cause a steadily increasing demand for high resolution quantitative magnetic characterization. Transmission electron microscopy (TEM) based measurements of the electron energy-loss magnetic chiral dichroism (EMCD) may serve as the needed experimental tool. To this end, we present a reliable and robust electron-optical setup that generates and controls user-selectable single state electron vortex beams with defined orbital angular momenta. Our set-up is based on a standard high-resolution scanning TEM with probe aberration corrector, to which we added a vortex generating fork aperture and a miniaturized aperture for vortex selection. We demonstrate that atom size probes can be formed from these electron vortices and that they can be used for atomic resolution structural and spectroscopic imaging – both of which are prerequisites for future atomic EMCD investigations.

## Introduction

For magnetic materials and in solid state physics in general, the orbital angular momenta (OAM) and spins of atoms and their mutual coupling are of surmounting importance, since due to the correlation of the OAM with the local electron density distribution and thus the crystal structure, they are intimately related to anisotropies in particular of the magnetic properties. The functions and properties of modern magnetic and/or spin-electronic devices are to an increasing extent due to their nanoscopic dimensions. Accordingly, these anisotropies may easily vary on length scales in the nanometer and sub-nanometer range such as at surfaces and interfaces or in the vicinity of defects. In a similar fashion, novel magnetic phenomena frequently go along with changes in or modulations of the arrangement of the local magnetic moments at likewise small length scales. E.g., the recently (re-) discovered skyrmions exhibit a complex toroidal arrangement of their magnetic moments^1–4^. Topological insulators gain their unique properties from the topological protection of surface states, which provide for spatially separated anti-parallel spin arrangements^5–8^. And even the properties of high performance bulk hard magnets depend to a large extent on the inter-granular magnetic coupling mediated by grain boundary phases, which are just a few atomic layers thin^9^. All these developments establish a steadily increasing demand to quantitatively determine spin and orbital magnetic moments with ultimate spatial – if possible atomic – resolution. The power and impact of a magnetic characterization tool that provides for atomic resolution was already convincingly demonstrated on surfaces using spin-polarized scanning tunneling microscopy (SP-STM)^10^.

Today, quantitative measurements of volumetric magnetic properties such as the magnetization, magnetic anisotropies, coercivities and switching fields are routinely accessible via, e.g., vibrating sample magnetometry or SQUID magnetometry, while details of the (global) spin structure and the element-specific average spin and orbital magnetic moments can be measured using, e.g., neutron diffraction or x-ray magnetic circular dichroism (XMCD). These methods, however, typically require access to large research facilities such as nuclear reactors or synchrotrons^11, 12^. Local measurements of the in-plane (IP) and/or out-of-plane (OP) components of the magnetization can be done using magneto-optical Kerr microscopy (IP, OP), magnetic force microscopy (IP, OP), Lorentz microscopy (IP) and electron holography (IP). Among those, the two latter, are conducted in transmission electron microscopes (TEMs). As a consequence, these techniques not only provide for high lateral magnetic resolution (usually down to 10 nm) but also offer the opportunity to correlate magnetic structures locally with the structure of the *identical* sample section. This opportunity to locally correlate properties and structure both quantitatively and with the highest possible resolution is essential to better understand modern materials and phenomena.

A novel method that combines quantitative magnetic information with high spatial resolution is electron energy-loss magnetic chiral dichroism (EMCD). EMCD was first proposed by Schattschneider *et al*.^13, 14^. Similar to XMCD, it allows for the element-specific (local) measurement of spin and orbital magnetic moments. However to date, the quality of the quantitative analysis is still limited by a relative poor signal-to-noise (S/N) ratio, and the spatial resolution of “classical” EMCD does not (yet) allow for atomic resolution^15–23^. Another proposal based on an imaging route and reconstruction of the probability currents might as well be able to obtain EMCD signals^24^. Atomic resolution EMCD may finally become feasible through the use of electron vortex beams (eVBs). eVBs have first been discussed theoretically by Bliokh *et al*.^25^ and were experimentally demonstrated by Uchida and Tonomura^26^ using stacked graphene layers, and by Verbeeck *et al*.^27^ and McMorran *et al*.^28^ with the use of holographic masks. Since such electron vortices carry discrete orbital angular momenta and can be easily focused down to sub-nanometer diameters, the use of eVBs for EMCD measurements may pave the way towards the quantitative determination of local magnetic properties with unrivalled lateral resolution in scanning transmission electron microscopes (STEM). The orbital angular momentum (OAM) L that is inherent to every vortex beam is due to an azimuthally increasing phase, which results in a spiraling wave front with a (phase) singularity in its axial center. Similar – and formally equivalent – to a polarized photon in XMCD, eVBs may interact with the spin system of a magnetic sample^29^. OAM-dependent electronic excitations from electronic (2p) core levels to partially unoccupied spin-polarized (3d) states lead to asymmetries (dichroism) in the electron energy loss spectra (EELS) in magnetic 3d transition metals, which are referred to as EMCD^13, 30, 31^. Hence, combining eVBs with EMCD promises to become a powerful tool to probe the local atomic magnetic moments, including their element-specific deconvolution into spin and orbital contributions, thereby providing insight into the atomic origin of, e.g., magnetic anisotropies or spin arrangements at the very atomic level. While it has been theoretically shown that for this combination, atom-sized probes are a prerequisite in order to be able to measure EMCD^32–34^, it may finally provide for magnetic measurements with true atomic resolution, if the remaining technical obstacles are overcome.

Today, eVBs can be generated with a variety of different methods that are all based on shaping the electron beam in the condensor lens system of the TEM by using holographic diffraction masks, phase plates, (switchable) magnetic needles, or aberration correctors. All these approaches have their individual advantages and disadvantages. The main problem of using holographic dislocation masks is, that all different vortex states (e.g. L = +1, L = 0, L = −1, and higher orders) are simultaneously present as focused beams at the sample. As an escape route to this scenario it was proposed to use holographic spiral masks, where the vortex states are dispersed in beam direction rather than across the sample^35^. Such beams were expected to have the advantage, that the desired vortex state can be addressed simply by properly defocusing the beam. It was, however, recently shown that even though the unwanted vortex states are largely defocused, they strongly contribute to the magnetic signal^36^. The use of phase masks does not suffer from this disadvantage, since here, only a single phase front is formed, which results in a clean, single vortex state^26, 37^. As a trade-off of this method, inelastic scattering in the mask always causes characteristic background signals in the EEL spectra. It will also at least be demanding to produce two sufficiently anti-symmetrical apertures in order to create identical L = 1 and a L = −1 beams, respectively. Beam damage of the aperture itself may be a further issue with this type of apertures. Similar difficulties arise using phase holograms in the fork aperture design^38–40^. Here, the beam is actually exchanging angular momentum with the aperture and its holder, which serves as an “infinitely heavy reservoir” leading to an enhanced efficiency of diffraction towards the desired vortex beam. Recently, Béché *et al*. have shown that a magnetizable needle that extends into an otherwise empty aperture may also provide for an azimuthally growing phase shift in the electron beam by virtue of the Aharanov-Bohm effect^41^. Upon switching the needle’s magnetization direction the helicity and OAM of the likewise created eVB change. Apparently due to some hysteresis, however, the position of the eVB changes slightly upon switching the OAM, which requires a manual alignment correction during the subsequent acquisition of images with varying OAMs. Finally, eVBs can be readily shaped from electron plane waves utilizing (probe) aberration corrector that are meanwhile available in many microscopes^42, 43^. Here, changing the OAM requires to frequently change the excitation of the corrector lens system, the feasibility of which is not yet explored.

In order to overcome the difficulties described above and to prepare a single, reliably user-switchable electron vortex probe with atomic size, we have devised and realized a novel optical setup. Using a holographic dislocation mask, we first produce a row of beams with different OAMs, which are – as usual – dispersed along a line perpendicular to the beam direction. By simply introducing an additional discriminator aperture into the optical path, all beams but the one, which carries the desired OAM, are blocked. A similar idea was recently described theoretically by Krivanek *et al*.^44^. We demonstrate, that the passing single vortex beam can then be focused down to angstrom-sizes achieving atomic resolution in STEM EELS, which is a critical pre-requisite for atomic resolution EMCD measurements.

## Results

**Figure 1:**
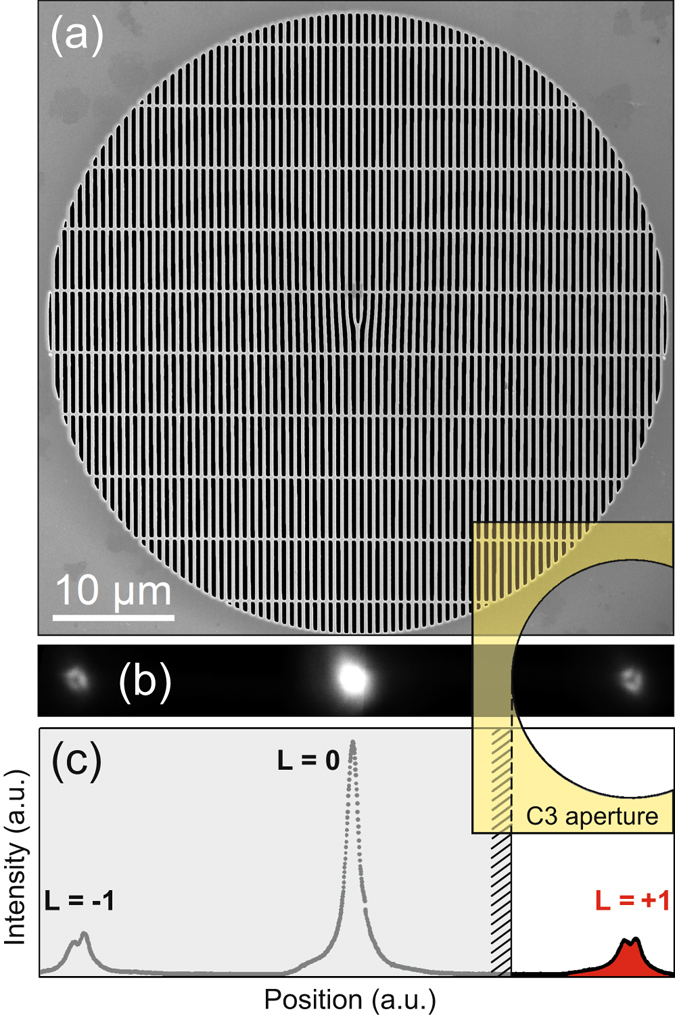
Generation of electron vortex beams with user-selected OAM using a fork-type aperture. (**a**) Scanning electron microscope image of a 50 µm dislocation aperture (places at the C2 aperture level). The horizontal ligaments are used as reinforcement of the 200 nm thick Pt foil. (**b**) Image of the electron probe at the sample. The position of the selected probe (L = +1) with respect to the discriminator aperture at the C3 aperture level is illustrated by the overlaid yellow structure. (**c)** Intensity profile across the probe (in arbitrary units). The signature donut shape of the outer vortices with |L| = 1 is reflected by a dip in the intensity distribution of these (partial) probes.

In the presented experiment, eVBs are produced by using holographic dislocation masks. Figure 1a shows a scanning electron microscope (SEM) image of such an aperture, which is installed right underneath the second condenser lens (C2 aperture) of the probe and image aberration corrected FEI Titan^3^ 80–300 microscope. Details of the mask preparation are given in the Methods section. The diameter of the dislocation mask is 50 µm, in order to provide for a sufficiently high beam intensity, while at the same time the maximum beam tilt still allows for a correction of the aberrations within the probe. At these diameters, however, additional reinforcement ligaments, which run perpendicular to the holographic grating, need to be implemented in the mask for mechanical stability. The number of ligaments was chosen to be as small as necessary in order to minimize the absorption of electrons. With the resulting mask it is warranted that almost 50% of the electrons that impinge on the aperture also pass it. Figure 1b shows a probe pattern as obtained at the sample plane. The intensity distributions of the diffracted beams clearly show the typical donut-like intensity profiles in their centers described by first order Bessel functions (Fig. S1 of the supplement shows an alternative representation of the beam in the diffraction plane). The vortex beam on the left side is defined as L = −1 and the diffracted beam on the right side is defined as L = +1. The undiffracted, central beam carries no OAM and is defined as L = 0. Note that since this image of the probe is obtained in STEM mode with switched-off diffraction, it is convoluted with the aberrations of the lower objective lens of the microscope (which appears as an additional triangularly shaped contrast). This imaging artefact is due to the fact that the image C_S_ corrector is not tuned in STEM mode (since it is usually not needed here). Hence as proven by the resolution of the STEM image, the true probe size is much smaller and of atomic size, thereby making sure that the different diffraction components of the probe, which carry different OAMs, are well separated and not overlapping. Analyzing the intensity in the vortex beams and neglecting higher orders, we find that the central beam carries roughly 60% of all electrons, while the two first order diffraction vortex beams (L = ±1) hold 20% each.

**Figure 2:**
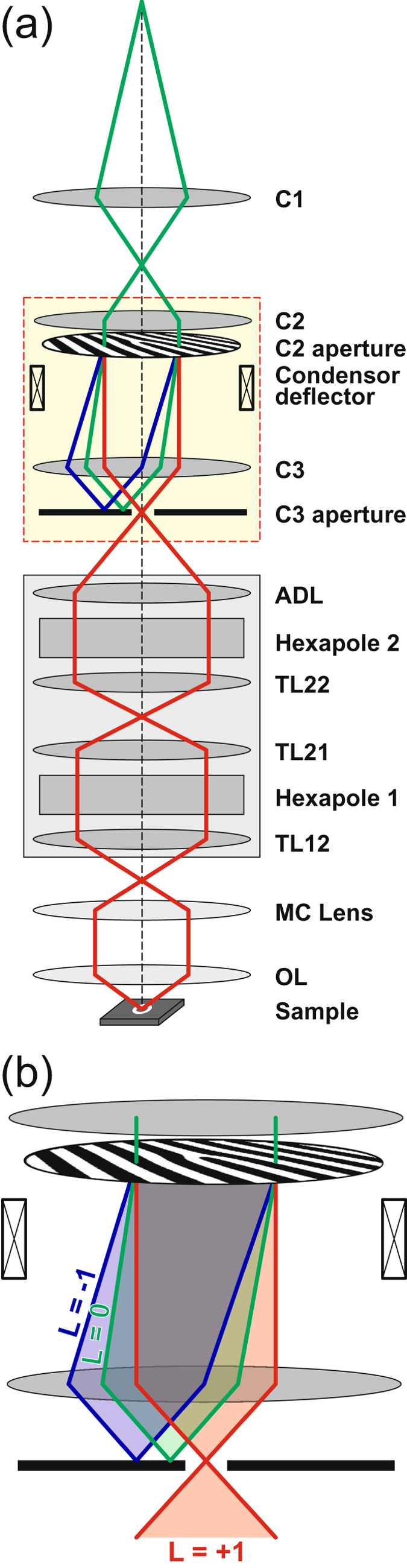
Electron optical setup for the generation of a switchable electron vortex state. (**a**) Schematic path of rays through the illumination lens system of the microscope. Here, Ci, ADL, TLxx, MC and OL denote the i^th^ condenser lens, the adapter and transfer lenses of the probe corrector (grey box), the mini condensor, and the objective lenses, respectively. (**b**) Magnification of the section between the 2^nd^ (C2) and 3^rd^ (C3) condensor lens (marked by a yellow rectangle in **a**), including the vortex aperture in the C2 aperture position. Blue, green, and red beams represent electron vortex beams with OAM of L = −1, L = 0 and L = +1, respectively.

Using the total probe shown in Fig. 1b with all diffracted beams for STEM imaging would lead to a largely blurred image, as a variety of spatially separated (partial) probes would concurrently scan different positions of the sample. In addition, it would be impossible to detect any EMCD signal, since the total OAM of the beam (i.e., the sum of the OAMs of all the partial probes), which interacts with the sample, is still zero. We have therefore introduced an unconventional optical condensor setup in order to block the unwanted portions of the diffracted beam. Figure 2 illustrates this concept. By focusing the 2^nd^ condensor lens (“C2”) to the image plane of the 1^st^ one (“C1”), the vortex aperture is illuminated with planar electron waves, whose transmitted partial beams interfere to form the diffracted vortex beams. By strongly exciting the 3^rd^ condensor lens (“C3”) the vortex beams are focused on the aperture plane of C3. Due to the periodicity of the grating in the vortex aperture of 640 nm, the diffraction angle is roughly 3 µrad, which provides for a lateral spacing of adjacent vortices of roughly 250 nm in the plane of the C3 aperture. At this C3 aperture position, a homemade discriminator aperture, which carries a single hole of 150 nm in diameter, is inserted such that the hole is centrally aligned to the optical axis. Using the C2 deflector coils, the set of vortices is then tilted until a single vortex beam with the desired OAM is placed in the hole of the discriminator (cf. enlarged illustration in Fig. 2b). Since the diameter of the hole is smaller than the lateral separation of the different vortices in the C3 aperture plane, all but the selected vortices are blocked. The likewise chosen vortex beam is then passed to the probe aberration corrector followed by the objective lens and finally focused on the sample.

In order to characterize the quality and feasibility of the introduced approach, SrTiO_3_ is chosen as a reference sample to prove the performance of this novel setup – both with respect to the achievable lateral resolution in STEM imaging and the capability to measure EEL signals from individual atomic columns with these focused eVBs. Here, SrTiO_3_ is particularly suited, since in [001] zone axis orientation, Sr and Ti atoms occur only in different atomic columns and are never mixed.

**Figure 3:**
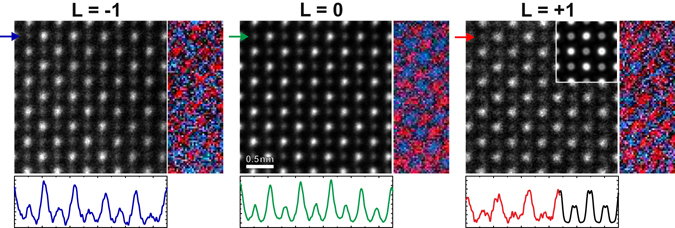
Scanning transmission electron microscopy and spectroscopy performance using single vortex beams on SrTiO_3_. All STEM images are acquired with electron probes that carry different OAMs (L = −1, L = 0, L = +1, from left to right) as provided by the novel optical setup. Right insets show false-color elemental maps of Ti (red) and Sr (blue) as obtained from EELS at the Ti-L and Sr-M edges. The lower panels show HAADF intensity profiles across the images at positions marked by an arrow. A further inset that is embedded in the HAADF image for the L = +1 eVB (right row) shows the result of an ADF simulation for a sample thickness of 20 nm and a source size broadening of the L = +1 beam of 30 pm. The intensity profile of this simulation is also included as a black line section in the lower panel.

Figure 3 shows high-resolution STEM images of a roughly 20 nm thin SrTiO_3_ sample oriented with the [001] zone axis parallel to the beam. The images are acquired with individually selected single eVBs that carry OAMs of L = −1, L = 0, and L = +1, respectively (from left to right). In order to account for residual sample drift, series with a total of 100 images each were acquired with short individual acquisition times. The image stack is upscaled by 200% and image alignment and scan-distortion compensation is performed using the SmartAlign software described in ref. 45. In addition, a median stacking algorithm has been used to remove statistical outliers from the image stack. As a result, the images shown in Fig. 3 represent the central images of stacks that were sorted by the pixel according to this median stacking. A comparison of the three STEM images in Fig. 3 reveals that the image obtained with the L = 0 probe has the highest intensity in both the Sr and Ti containing columns, which is due to the higher intensity of this central beam (cf. Fig. 1). The spatial resolutions as determined from the outmost visible reflections in the Fourier transforms of the images are 87 pm (SrTiO_3_ (024)) and 123 pm (SrTiO_3_ (031)) in the images acquired with eVBs carrying OAM’s of L = 0 (central image) and L = ±1 respectively. This proves that for the given sample and orientation atomic (column) resolution is warranted also when images are acquired with electron probes, which carry orbital angular momenta, as generated with the presented optical setup. The reduced spatial resolution of the vortex beams as compared to the L = 0 beam is due to their larger FWHM^33^.

Like in all probe based methods, the STEM image strongly depends on the shape of the probe wave function. In the case of a normal STEM probe the probe wave function is represented by an airy disk resulting in Gaussian intensity profiles of the atomic columns in the STEM image. For vortex beams, the wave functions are described by Bessel functions. As a consequence, atomically resolved STEM images acquired with such beams should as well show the fingerprint of the probe’s donut-like intensity distribution, and the intensity distributions around a single atomic columns should deviate from a Gaussian distribution.

This deviation can indeed be observed in the experimental STEM images. Upon closely examining in particular the STEM image obtained from the eVB with the L = +1, the donut-type signature of the imaging vortex beam can be recognized at both the elemental Sr and the mixed Ti-O atomic columns, respectively. It manifests itself through dips in the image intensity as revealed by cross-sectional intensity profiles along single rows of atoms (cf. line profiles in Fig. 3). In the simulated ADF STEM image of the L = +1 probe (cf. inset in the right upper corner of the top STEM image), the dip turns out to be smaller and less pronounced on the Sr as compared to the Ti containing columns. Residual aberrations, thermal and athermal phonons, drift and a slight defocus may, of course, easily smear out this donut-type signature of the vortex. As a consequence, and although the intensity distributions of almost all columns indicate the presence of a dip, there are only a few rows, where these features are strong. Recent ADF simulations on the interaction of a vortex probe with bcc Fe as function of the thickness and the source size broadening have shown that also the source size broadening may effectively impede the visibility of the donut pattern in the STEM image^46^. In the performed ADF simulations, the ring-shaped intensity profile is visible for all examined sample thicknesses up to 62 nm, when source size broadening is neglected. The pattern, however vanishes for source size broadenings larger than 40 pm. The inset in Fig. 3 shows the result of an ADF simulation for a sample thickness of 20 nm and a source size broadening of 30 pm that is added to the diffraction limited probe size of roughly d_Diff_ = 0.61 * λ/α = 57 pm. This simulation yields the best agreement with the experimental image. Unfortunately, a more thorough quantitative analysis is impeded by the still high noise level in the experimental images due to the low currents in the selected eVBs (I_0_ = 40 pA, I_±1_ = 13 pA).

In order to substantiate that the proposed electron-optical setup will enable future EMCD measurements with up to atomic resolution, it is yet to be shown that our approach also offers the possibility to acquire EEL spectra with the same resolution. This capability to measure EELS from individual atomic columns with accordingly generated EVBs is highlighted by mapping the EEL intensities at the Sr-M and Ti-L edges, respectively. The results of these measurements are included as false-color insets at the bottom right corners of the ADF STEM images in Fig. 3 for all three types of probes (with red and blue representing Sr and Ti, respectively). Like in the STEM images, the strongest elemental intensity is again obtained using the central beam with OAM L = 0. Also in the case of the vortex beams L = −1 and L = +1, however, clearly atomically resolved Sr and Ti maps are obtained. Similar to the HRSTEM images, also the EELS data suffer from low probe currents.

Following the discussion by Thersleff^47^ on the signal-to-noise ratio, the counts n_e_ needed to detect an EMCD signal with the 5σ Rose criterion are:

$${n}_{e} > \frac{25\cdot (1+b)}{{r}^{2}},$$

with b and r denoting the signal-to-background ratio (SBR) and the expected strength of the EMCD signal, respectively. Evaluating the EEL spectra for the Ti-L edge acquired on SrTiO_3_ with the L = +1 vortex beam, the SBR is found to be b = 1.43. Assuming an EMCD strength of r = 0.03, 67500 counts are needed. For the given Ti spectra, that would correspond to a summation over 19 atomic columns thereby providing for an ensemble average over that area. And while statements on the properties of a single atomic column cannot yet be made at this stage, the freedom to choose an arbitrary averaging pattern, e.g., a pattern of 19 × 1 columns, allows to conduct measurements with a resolution of down to a single atomic *layer* at least in one direction. Furthermore, walking window techniques may be used to account for the continuously varying (due to their exchange stiffness) rather than abruptly changing magnetic moments, e.g., across a domain wall. Hence, even with the present SNR-related limitation, the measurement of magnetic profiles with up to single atomic layer resolution comes within reach. In order to reach quantitative atomic resolution EMCD on single atomic columns and to overcome signal-to-noise ratio problems in future works, smart acquisition strategies and new, efficient low noise detectors might be needed. Hence, the use of a holographic diffraction mask in combination with a possibility to user-adjust the OAM of the electron probe may pave the way to locally measure magnetic properties with atomic (column) resolution.

## Discussion

In 2014, Schattschneider *et al*. had raised the question: “Is magnetic chiral dichroism feasible with electron vortices?”. At that time – given the then available vortex probe dimensions – the question could only be negated. Simulations had rather suggested that it should only be possible to measure EMCD with eVBs, if a single vortex is focused onto an individual atomic column^32–34^. With our here proposed setup and similarly with an alternative approach by Béché *et al*.^41, 48^ it is meanwhile possible to provide for atom sized electron vortex probes that are expected to indeed enable atomic resolution EMCD measurements. While Béché *et al*. use the magnetic stray flux of a magnetizable needle to generate single electron vortex beams, the present approach makes use of a holographic diffraction mask to form a whole set of vortices with finite orbital angular momenta. And although in the latter, an additional aperture is needed to select the wanted eVB, our approach has the advantage that due to the symmetry of the aperture, beams of opposite OAM, e.g., with L = −1 and L = +1 are truly equal (except for their OAM). Using this setup, it is even possible to observe – though faintly – the signature of the imaging vortex beam directly in an experimental atomic STEM image, just as expected due to the convolution of the probe with the structure of the sample. The novel setup does not require for any hardware modifications of the microscope. Solely, the vortex and discriminator apertures, which can both be easily home-made with a state-of-the art FIB, need to be inserted into the appropriate condensor aperture holders. The OAM selection (by means of the C2 condensor deflector coils) is easy, reproducible and can be controlled from within the readily available graphical user interface (GUI) of the microscope (at least in case of FEI Titan microscopes). The quality of the STEM images and EELS-based elemental maps, which both provide atomic resolution, promise to open the door for future quantitative measurements of magnetic properties with ultimate spatial resolution and their local correlation with structural features at the very same position within the identical sample. Further potential applications of the presented electron-optical configuration, making use of the unique possibility of the selection of higher order vortices, are “spiral phase contrast” edge enhancement^49^ for e.g. biological samples and out-of-plane magnetic mapping^50^.

## Methods

For the preparation of the Pt apertures, a 200 nm thin Pt layer was sputtered onto a NaCl crystal. The thin Pt foil was then extracted by desolving the NaCl in water. Afterwards, the Pt foil, which is used for the creation of the dislocation mask was taken to fill the 200 µm hole in a commercially available Pt aperture.

The dislocation aperture and the 150 nm small C3 aperture are fabricated with a FEI Helios 600i FIB working at 30 kV. As a template for the FIB cutting process, a binary image was used. This binary image was created by the interference of two waves: a tilted plane wave and a wave with an azimuthally growing phase of up to 2π. The binarization was chosen to result in the same amount of black and white pixels inside the circular aperture.

The dislocation aperture is inserted at the C2 aperture position in the condenser system of a FEI Titan^3^ 80–300 image and probe corrected electron microscope operating at 300 kV. The dislocation aperture gives rise to an angular separation of 3 µrad between the different vortex beams L = 1, L = 0, L = −1. The C2 lens and C3 lens are tuned such that a cross-over is made exactly at the C3 aperture plane. The vortex beam is selected by a 150 nm aperture in this plane.

The selected vortex beam is transported by the ADL lens trough the probe corrector (DCOR) to the objective lens, and the objective lens de-magnifies the vortex beam to an atomic sized probe at the specimen. The opening angle of this probe is set by the diameter of the dislocation aperture, and it can be tuned (within a range of about a factor three) by slightly (de-)exciting the C3 lens and retuning C2 in order to refocus the intermediate image of the probe back into the C3 aperture. The probe is coarse-focused on the specimen by tuning the ADL lens, and the probe is fine-focused by tuning the objective lens.

The aberration correction of the DCOR is done in the normal STEM settings with a convergence angle of 30 mrad using the tableau method in the DCOR user interface. Subsequently the condensor system is set up for the vortex selection (C2 and C3 lens excitation) and the ADL lens is used to refocus the beam. The beam is shifted using the pre-corrector beam tilt and beam shift. Residual low order aberrations (coma and astigmatism) are fine-tuned using the ronchigram of amorphous carbon.

HRSTEM images are acquired using a high angle annular dark field (HAADF) detector with a camera length of 91 mm (collection angle 49.5 mrad – 200 mrad) and a convergence semi-angle of 22.6 mrad. For the acquisition of the EEL spectra, a GATAN Tridiem 863ER image filter with a dispersion of 0.5 eV/pixel is used. The spectra are measured with a collection semi-angle of the spectrometer of 61.9 mrad. An average sample thickness of roughly 20 nm was determined by using low loss EELS (low loss EELS data shown in Fig. S3 in the Supplementary information).

Prior to the non-rigid registration an upscaling by 200% using bilinear interpolation of the images in the stack, in order to achieve digital super resolution, is performed. To remove outliers in the image stack, a median sorting algorithm has been used after drift correction. The algorithm sorts every pixel in the stack by its intensity value. The central image of the stack (median) is then chosen for further analysis.

HAADF simulations were done using a real space multislice method^49^ within a frozen phonon approximation. The snapshots of the vibrating crystal were generated with classical molecular dynamics code LAMMPS^51, 52^ using interatomic potentials optimized for SrTiO_3_^53^. This approach considers the correlated motion of atoms and phonon dispersion relations to a high accuracy. Acceleration voltage, convergence and collection angles were set according to experiment. For comparison with experiment, the simulations were run for a range of thicknesses from 2 to 60 nm and various source size broadenings were modelled using a Gaussian blur^54, 55^.

## Electronic supplementary material

Supplementary Information
