# Supplementary material for: Atom size electron vortex beams with selectable orbital angular momentum
Source: Sci Rep. 2017 Apr 19;7:934. doi: 10.1038/s41598-017-01077-9 (PMC5430437; doi:10.1038/s41598-017-01077-9)
Supplement: Supplementary file 1 — Supplementary Information [file 41598_2017_1077_MOESM1_ESM.pdf]

Supplementary Information

Atom size electron vortex beams with selectable orbital angular momentum

Darius Pohl[1], Sebastian Schneider[1,2], Paul Zeiger[3], Ján Rusz[3], Peter Tiemeijer[4], Sorin Lazar[4], Kornelius Nielsch[1,5] and Bernd Rellinghaus[1].

1 IFW Dresden, P.O. Box 270116, D-01171 Dresden, Germany.
2 TU Dresden, Institute for Solid State Physics, D-01062 Dresden, Germany.
3 Uppsala University, Department of Physics and Astronomy, SE-752 37 Uppsala, Sweden.
4 FEI Company, PO Box 80066, 5600 KA Eindhoven, The Netherlands.
5 TU Dresden, Institut für Werkstoffwissenschaft, D-01062 Dresden, Germany.

Correspondence and requests for materials should be addressed to D.P. (email: d.pohl@ifw-dresden.de)

Visualization of vortex beams

The main characteristic of an electron vortex beam, is its azimuthally growing phase (s. fig S1). The most intuitive way of visualizing the vortex is to look at its far-field on a screen, where a donut-like intensity distribution is found, stemming from the modulus square of the Bessel function. However, one crucial point is the phase singularity in the center of the vortex beam. This singularity manifests itself not only in the far-field of the vortex beam, but also in the modulus of the wave function itself. In the experimental setup, vortex beams are generated by a holographic mask in the condenser lens system of the microscope and are focused as probes onto the sample. In STEM mode, a diffraction disk (or in the absence of a sample, the image of the limiting aperture) is visible on the screen. In the case of single electron vortex beams, here a central dip of intensity can be found for the L = ±1 beams, whereas it is absent in the case of the L = 0 beam (cf. fig. S1).

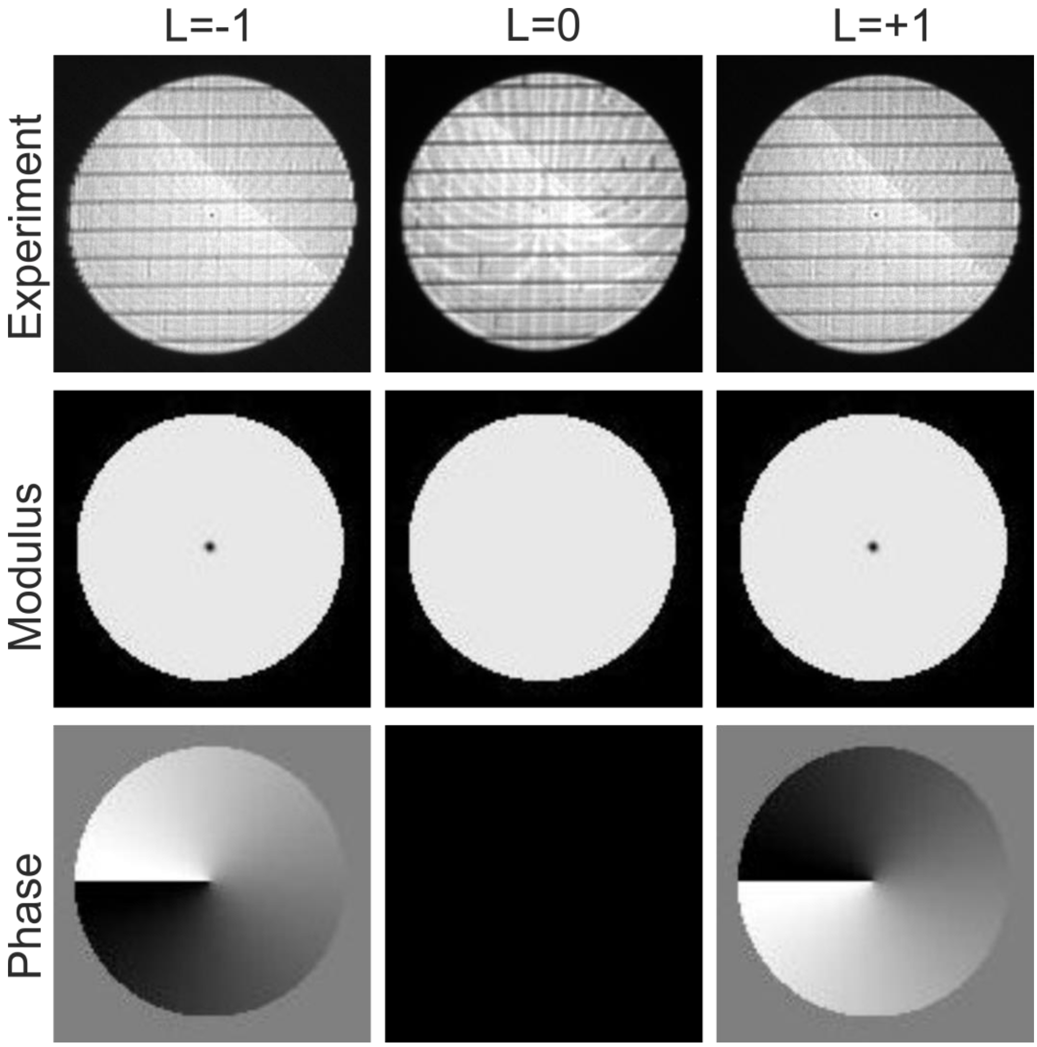

Suppl. Fig. S1: **Single beam diffraction disks.** Experimental diffraction disks show pronounced intensity dip in the center for the L = +1 and the L = -1 beam (absent in the L = 0 beam). Simulated wave functions show the same intensity dip in the case of the vortex beams, caused by the phase singularity in the center.

HAADF simulations

HAADF simulations using a vortex wave function as a probe are performed, for comparison with experiment, for a range of thicknesses from 2 to 60 nm and various source size broadenings which are modelled using a Gaussian blur (0 - 200 pm). A summary for the most interesting range is given in fig. S2. The donut-like intensity disappears after source size broadening of about 40 pm (FWHM).

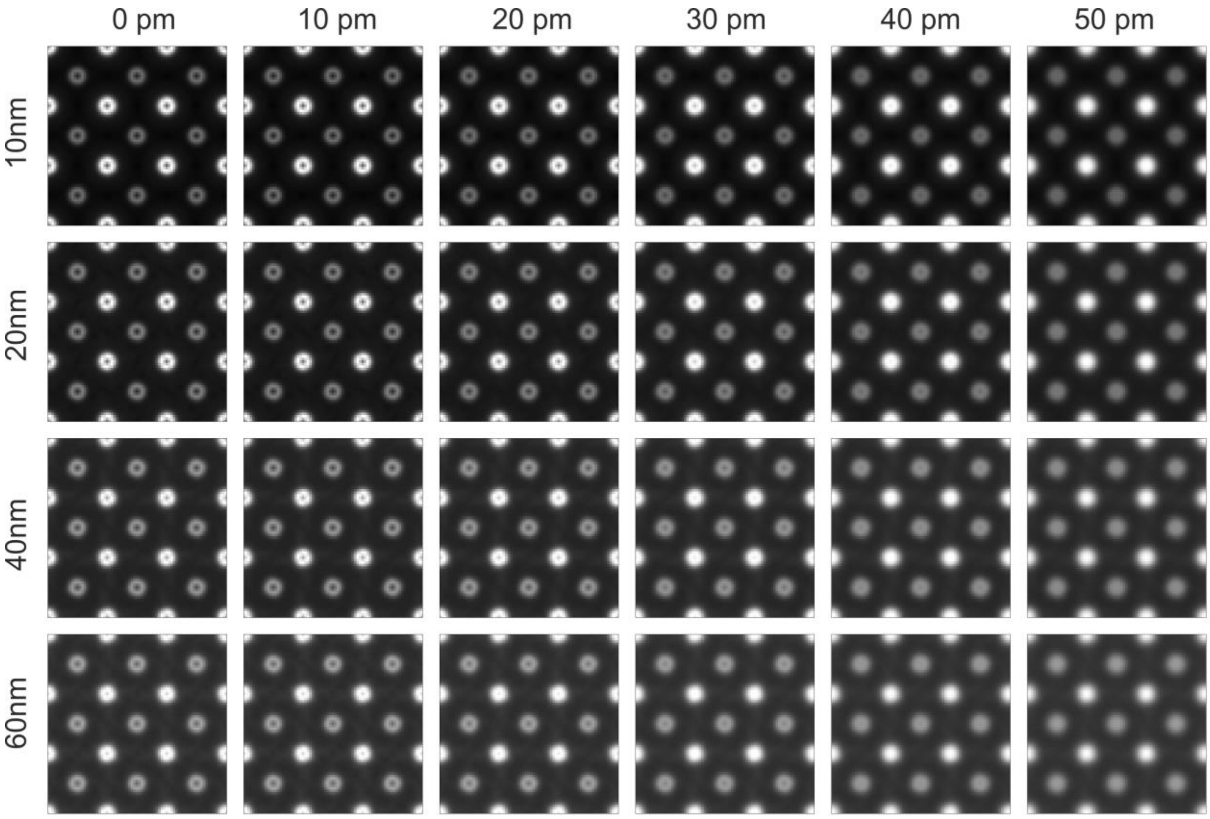

Suppl. Fig. S2: **HAADF simulation of SrTiO3 imaged with an L = +1 vortex beam.** Each panel shows 3x3 unit cells of SrTiO3 in (001) zone axis orientation, calculated at room temperature. Columns from the left to right show source size broadening of 0 - 50 pm with steps of 10 pm. Rows from top to bottom show calculations for thickness 10 nm, 20 nm, 40 nm and 60 nm.

Thickness determination using Low Loss EELS

The sample thickness of the investigated area was determined by low loss EELS. Analysis of the low loss spectra (L = 0) gave an average absolute thickness of 19.11 nm. Fig. S3 shows the low loss spectra for all three beams (L = 0, L = ±1)

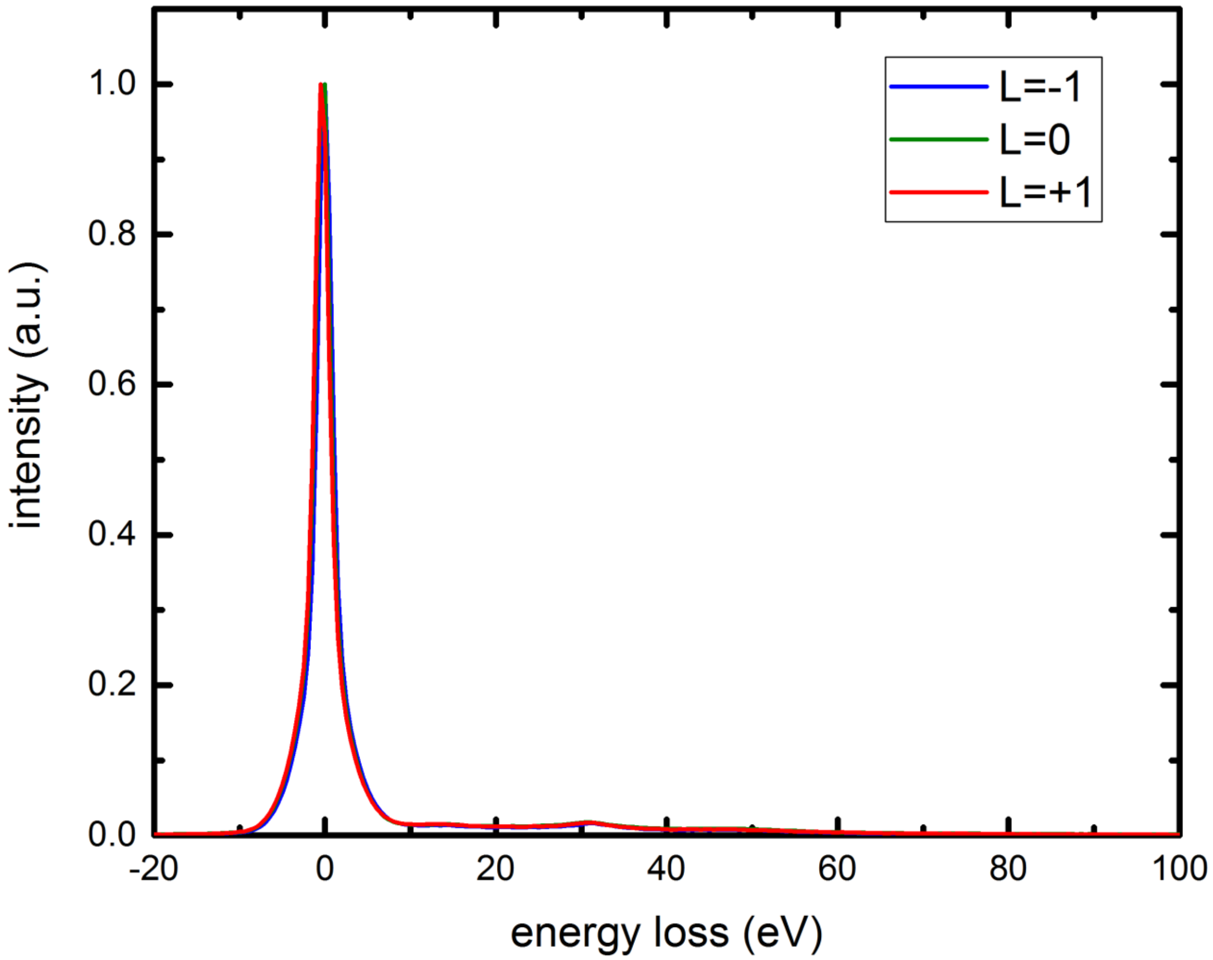

Suppl. Fig. S3: **Low loss EELS data (normalized) of the investigated $SrTiO_3$ sample acquired with the eVBs (L=0, L = ±1).**

Further image analysis of fig. 3

An average background subtraction filter [S1,S2] has been applied to the images of fig. 3, to more clearly visualize the differences between the L=0 and the e.g. L=+1 beam. Fig. S4 shows the images for the L=0 and the L=+1 beam (gamma value set to 0.25), where now mainly the Sr columns are visible. For selected atom columns (numbered 1-5), zoom in images are shown on the right. (i) We observe a clear increase of the diameter of the imaged atom columns, as expected from the initially broadened probe in the case of using eVBs as compared to using a L=0 beam. (ii) In addition, and unlike the image obtained with L=0 the intensity distribution obtained for individual atomic columns becomes inhomogeneous upon using the L=±1 beam. Frequently, we even observe the formation of a central drop in intensity (see magnified images on the right), as expected from the simulated ADF images. Apparently, however, the visibility of that dip crucially depends on the degree of blurring like e.g. the alignment within the individual atomic columns, and can thus not always be clearly identified. As a consequence, averaging of all columns results in a loss of the dip-like feature.

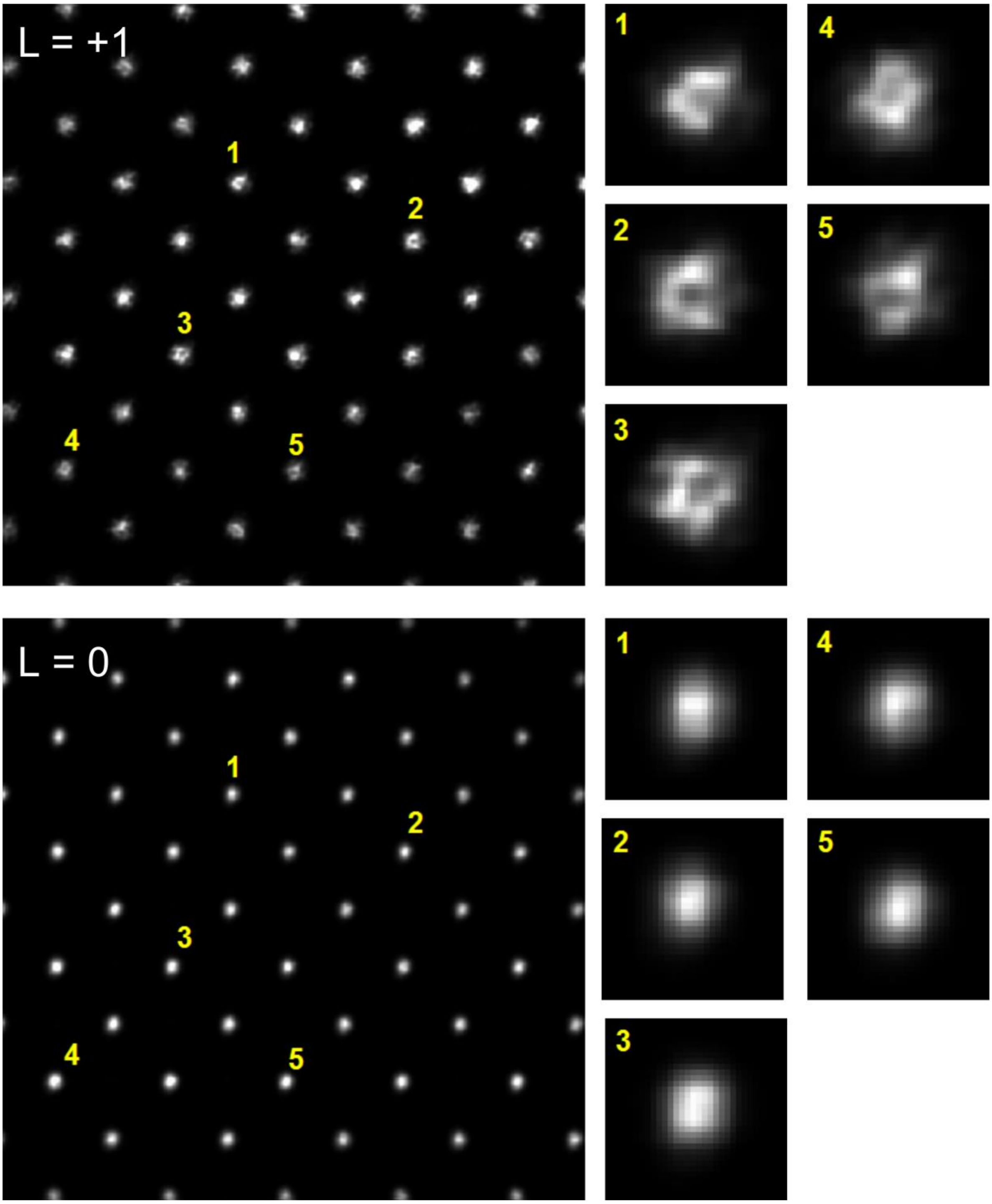

Suppl. Fig. S4: **Average background subtraction filtered HRTSEM images of fig. 3 (L=+1 and L=0 beam).** Right images show zoom in to the numbered positions.

Signal-to-noise ratio estimation

The relative strength of EMCD signal is called r. The edge counts are n_e and the power-law background counts are n_b. For quantitative analyisis using the sum rules not the single bins but rather the areas are used – a sum over the whole L_3 edge. To have a reasonably precise estimate of the EMCD counts we aim for 5σ Rose criterion:

$$r \cdot n_e > 5 \cdot \sigma \quad (1)$$

Where

$$\sigma = \sqrt{n_e + n_b} \qquad (2)$$

To simplify, the qualitative assumption that n_b is proportional to n_e is made (at a given experimental geometry and fixed sample thickness) $n_b = b*n_e$. Then (1) becomes

$$r \cdot n_e > 5\sqrt{n_e(1+b)} \qquad (3)$$

$$\sqrt{n_e} > 5\,\frac{\sqrt{1+b}}{r} \qquad (4)$$

$$n_e > \frac{25 \cdot (1+b)}{r^2} \qquad (5)$$

For the estimate in the manuscript, the Ti-L edge was summed over 18eV at the atomic column of an area of 3x3 pixel.

References

[S1] Kilaas, R., Optimal and near-optimal filters in high-resolution electron microscopy, J. Microscopy **190,** 45-51 (1997).

[S2] Mitchel, D. HRTEM Filter, http://www.dmscripting.com/hrtem_filter.html (2014), (Date of access:01/11/2016)
